# Photoinitiated Marangoni flow morphing in a liquid crystalline polymer film directed by super-inkjet printing patterns

**DOI:** 10.1038/s41598-019-38709-1

**Published:** 2019-02-22

**Authors:** Issei Kitamura, Kazuaki Oishi, Mitsuo Hara, Shusaku Nagano, Takahiro Seki

**Affiliations:** 1 Department of Molecular and Macromolecular Chemistry, Graduate School of Engineering, Nagoya University, Furo-cho, Chikusa, Nagoya 464–8603 Japan; 2 Nagoya University Venture Business Laboratory, Furo-cho, Chikusa, Nagoya 464–8603 Japan

## Abstract

Slight contaminations existing in a material lead to substantial defects in applied paint. Herein, we propose a strategy to convert this nuisance to a technologically useful process by using an azobenzene-containing side chain liquid crystalline (SCLCP) polymer. This method allows for a developer-free phototriggered surface fabrication. The mass migration is initiated by UV-light irradiation and directed by super-inkjet printed patterns using another polymer on the SCLCP film surface. UV irradiation results in a liquid crystal-to-isotropic phase transition, and this phase change immediately initiates a mass migration to form crater or trench structures due to the surface tension instability known as Marangoni flow. The transferred volume of the film reaches approximately 440-fold that of the polymer ink, and therefore, the printed ink pattern acts as a latent image towards the amplification of surface morphing. This printing-aided photoprocess for surface inscription is expected to provide a new platform of polymer microfabrication.

## Introduction

The free surface plays an important role in the motions of fluid materials. When a surface tension difference exists at the free surface, flow motions are induced, widely known as Marangoni effect^1^. Such flow progression are ubiquitous in nature, and can be seen in everyday life^1,2]^. For polymer film materials with a moderately high viscosity^3^, this effect may deform the film surface, and then the deformed shape is fixed after solvent evaporation or vitrification by cooling. In general, such surface distortions and defects in polymer films are serious nuisances, preventing smooth film coatings. Examples can be found in crater formation during the painting process^4–6^, striation defect generation in spincast films^7^, and difficulties in droplet shape control in inkjet printing^8–11^.

If the flow-induced deformation of polymer surfaces can be controlled as designed, this will provide a powerful tool for surface microfabrication. In this regard, Ellison and coworkers^12–16^ have proposed the pioneering concept of arbitrary inscription of polymer surfaces based on the Marangoni flow via photopatterning on the free surface. A melt-flow is induced according to photopatterns upon annealing above the glass transition temperature (*T*_g_) of the base polymer. Singer *et al*.^17,18^ have studied a focused laser-induced Marangoni de-wetting for amorphous polymer films, and Elashnikov *et al*.^19^ have demonstrated a laser-heating-directed Marangoni flow on a dye-doped poly(ethyl methacrylate) film. Along the same line, Ubukata *et al*.^20^ have also observed a relevant mass transfer phenomenon of an amorphous film, which is discussed from a different viewpoint of material diffusion affected by photochemical crosslinking.

On the other hand, in the systems of side chain liquid crystalline polymer (SCLCP) films, it has recently been indicated that the free surface plays a significant role in the alignment control of side chain mesogens^21–28^. The photoalignmnt of liquid crystals are conventionally performed from solid substrate surfaces^29–33^, however, it has recently been unveiled that photoalignment from the free surface is particularly effective in SCLCP film systems. We have proposed procedures of photoalignment of mesogens triggered from a photoresponsive skin layer existing at the free surface of an optically inert SCLCP film by irradiation with linearly polarized light (LPL)^21,22,25^. Kawatsuki *et al*.^26–28^ have also demonstrated the control of mesogen orientation from the free surface using different types of photoreactive unit. The top skin layer can be prepared by surface segregation of a surface active photoresponsive polymer^21,22,25^ or local deposition by inkjet printing^22,27,28^.

**Figure 1:**
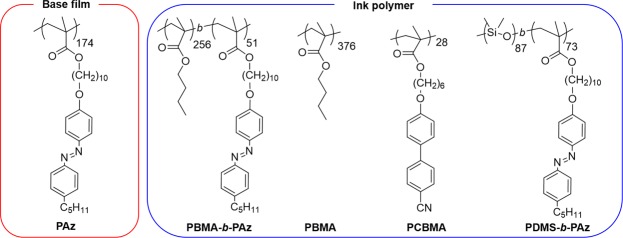
Chemical structures of polymers used in this study.

Here, we report a new free surface-mediated optical effect exhibiting large and instantaneous surface morphing directed by super fine-inkjet printing patterns in a photoresponsive SCLCP film. The mass migration motion due to the Marangoni flow was initiated by a photoinduced phase transition^34^ in an azobenzene-containing SCLCP film (poly[4′-10-(methacryloyloxy)decyloxy-4-(heptylpheynylazo)phenyl], (PAz)) in Fig. 1). Poly(butyl methacrylate)-*block*-PAz (PBMA-*b*-PAz) was mainly used as the ink, and other polymers were also used for different purposes (Fig. 1). The resulting surface morphology was obtained as a dip in the center and embossments in the periphery regions, a characteristic feature of crater formation by contaminations in paint. The superfine inkjet-printing drawing provides spatially designed micrometer-scaled heterointerfaces without damages to the surface of the base polymer film. The volume of transferred polymer reaches a level of a hundred times that of printed polymer ink. In this sense, the printed ink pattern on the surface works as a latent image, and UV irradiation corresponds to a dry development process, leading to the surface microfabrication.

## Results

### Polymer Materials

All polymers used in this study were synthesized in this laboratory^21,22,24,35^, and the chemical structures are displayed in Fig. 1, together with the characterization data. Differential scanning calorimetry (DSC) curves are shown in Supplementary Fig. 1. The isotropization (smectic A to isotropic) temperature (*T*_iso_) of PAz was 116 °C. Diblock copolymers composed of the PAz block and an amorphous coil block (PBMA-*b*-PAz and poly(dimethylsiloxane)-*block*-PAz (PDMS-*b*-PAz)) show essentially identical thermophysical properties, indicating the formation of a microphase separated structure. The contact angles of a glycerol droplet (*θ*_gly_) (*γ*_gly_ = 63 mN m^−1^ at 25 °C) are summarized in Table 1 for the measure of surface tension of the polymer film surface. Glycerol was used based on the requirement of high temperature measurements at approximately 100 °C. The contact angles of a water droplet (*γ*_w_ = 72 mN m^−1^ at 25 °C) on these polymer films at 25 °C are indicated in Supplementary Fig. 2 for reference.

**Table 1:** Characterizations of polymers.

Polymer	*M*_n_ (*M*_w_/*M*_n_)	Degree of polymerization	Thermophysical Properties/°C	Contact angle of glycerol (*θ*_gly)_/deg			Reference(s)
				25 °C	90 °C	130 °C	
PAz	8.6 × 10^4^ (1.54)	174	g (55) sm C (87) sm A (116) iso	102.5 ± 0.9	103.2 ± 0.7	93.3 ± 1.8	^21^
PAz (UV)				89.1 ± 1.8	85.1 ± 1.6	83.7 ± 1.5	
PBMA-*b*-PAz	5.4 × 10^4^ (1.10)	256 (PBMA), 51 (PAz)	g (54) sm C (83) sm A (115) iso	93.3 ± 1.0	102.0 ± 1.1	101.0 ± 2.5	^21, 22^
PBMA	5.3 × 10^4^ (1.16)	376	g (ca.20)	93.0 ± 0.8	101.0 ± 1.1	100.7 ± 1.5	^21^
PCBMA	1.0 × 10^4^ (1.13)	28	g (53) sm A (115) iso	84.9 ± 1.2	84.2 ± 1.0	80.3 ± 1.4	^22, 24^
PDMS-*b*-PAz	5.6 × 10^4^ (1.20)	87 (PDMS), 73 (PAz)	g (53) sm C (81) sm A (112) iso	110.0 ± 0.7	116.7 ± 1.1	102.6 ± 1.8	^35^

PAz (UV): under UV light irradiation, g: glass, sm: smectic, iso: isotropic.

### Photoinitiated mass migration

**Figure 2:**
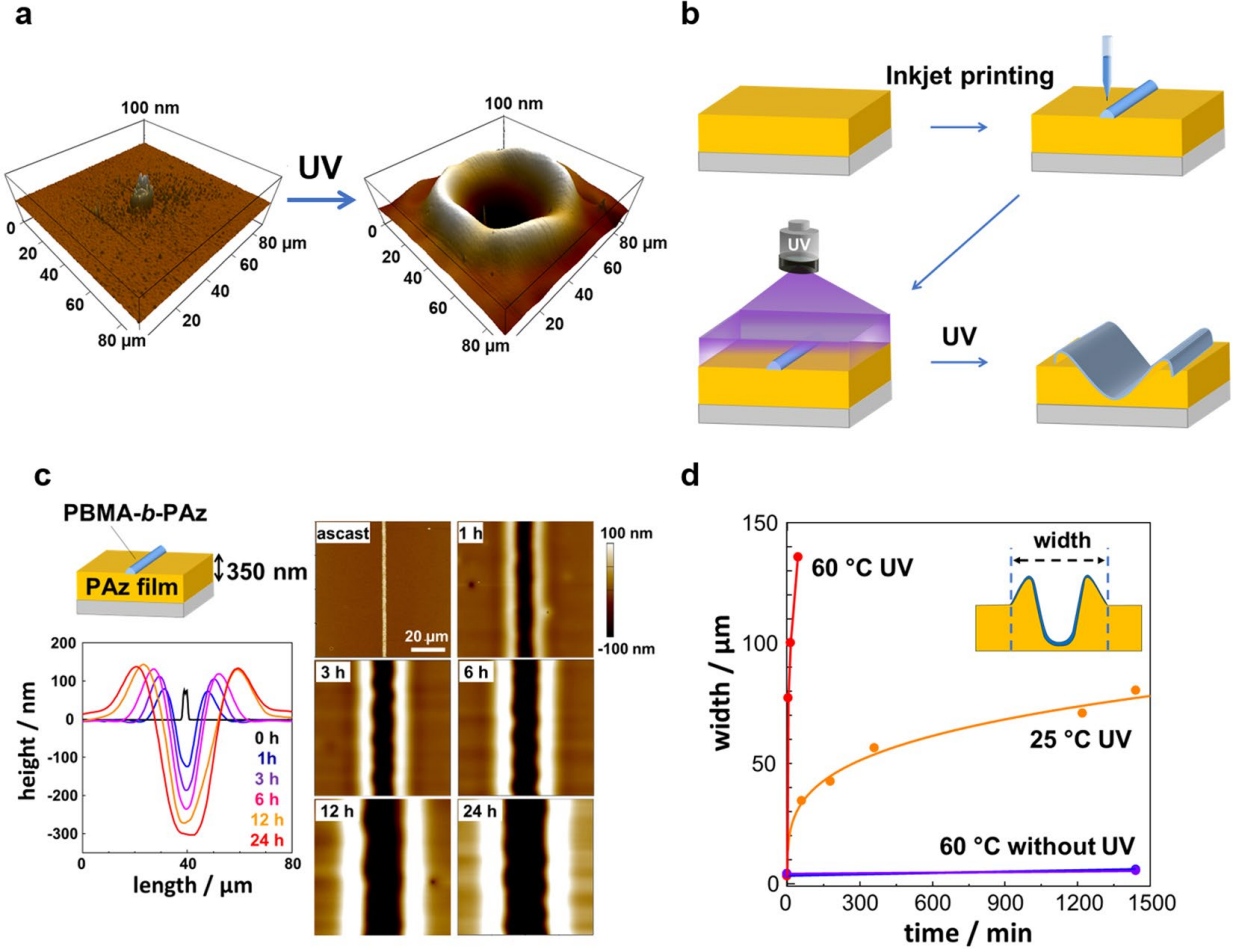
Photoinitiated mass migration. (**a**) AFM images of a printed dot on a PAz film (left) and crater formation after UV-light irradiation (right). (**b**) Schematic of the series of procedures adopted in this work. (**c**) Topographical AFM images demonstrating the mass transfer of a PAz film with a thickness of 350 nm under UV-light irradiation (10 mW cm^−2^) at 25 °C, and changes in the cross-sectional profile obtained from the AFM data. (**d**) Changes in the linewidth with time without UV irradiation at 60 °C, and with UV irradiation (10 mW cm^−2^) at 25 °C and 60 °C.

Typical examples of UV-light-initiated surface morphology induction are displayed in Fig. 2. A spincast film of PAz was prepared (typical film thickness: approximately 350 nm), and, onto this film, a dot of a block copolymer (PBMA-*b*-PAz) dissolved in a mixed solvent (0.5% by weight) of *o*-dichlorobenzene and *N*-methyl-2-pyrrolidone (30:1 by volume) was ejected using a superfine-inkjet printing apparatus. This apparatus enabled ejections at a sub-femto liter level. The dimensions of the dot were 5 μm in diameter and 100 nm in height. In the conditions employed, the solvent was nearly evaporated during the flight from the inkjet nozzle to the PAz film surface, suggesting that the solvent hardly disrupted the PAz film surface. When the dot (Fig. 2a, left) was irradiated with UV light at 365 nm at 60 °C, a mass transfer occurred to form a crater (Fig. 2a, right). Such a dip formation surrounded by a rim is a characteristic of crater defects in paint coatings originating from a contamination existing on the surface^4–6^.

Having this result, line patterns were hereafter routinely drawn under the same conditions by continual ejection for experimental and analytical conveniences (a scheme of the procedures is shown in Fig. 2b). The mass migration occurred essentially only in a one-dimensional manner, namely perpendicular to the printed line, leading to the reliable and reproducible evaluation of mass transfer process from the linewidth. After a line of PBMA-*b*-PAz was drawn by inkjet printing, the whole surface was uniformly irradiated with UV light at a constant temperature. The widths of the printed line were typically 5 μm with a 70 nm height at the center.

Figure 2c shows topographical atomic force microscopy (AFM) data of the surface morphology and profile with time under irradiation with UV light (10 mW cm^−2^) at 25 °C. Under these conditions, the azobenzene unit was isomerized to the cis-form and reached the photostationary state (cis content: 76%, see Methods) in 20 s. As shown, the mass migration occurred slowly over a range of hours to form a trench and ridges on both progression sides. The linewidth defined as the distance between the two ridge edges was monitored as a function of time (Fig. 1d). Compared to the data at 25 °C, the migrating motion was prominently accelerated at 60 °C. Contrastingly, the linewidth was hardly changed when the UV light was not shone. It is well known that an isothermal liquid crystalline to isotropic phase transition is induced by UV-light irradiation as a result of the accumulation of cis-azobenzene^37^. This phase transition leads to a liquefaction of such polymers^36–38^. Actually, our previous work has indicated that PAz exhibits the light-induced phase transition (Supplementary Fig. 3)^39^.

Steady flow viscosity measurements were made for PAz using a cone-plate rotational rheometer. The irradiation was achieved from the bottom of the shear cell in the apparatus. Large differences in shear viscosity were observed between the irradiated and non-irradiated samples depending on the shear rate in the dark and under UV irradiation (10 mW cm^−2^) (Supplementary Fig. 4). However, the data observed under UV-light irradiation here does not reflect the actual viscosity of the UV photostationary state because the sample thickness (50 μm (cone center) to 480 μm (periphery)) was far beyond the light penetration depth (below 1 μm). The photoisomerization to cis-form of azobenzene occurred partly only on the surface of the illuminated side. Nevertheless, the apparent viscosity change was evident, namely, the viscosity was substantially lowered under the UV irradiation conditions at low shear ratios (Supplementary Fig. 4b). This significant reduction in viscosity of the PAz base film should be ascribed to the induction of the linewidth evolution.

**Figure 3:**
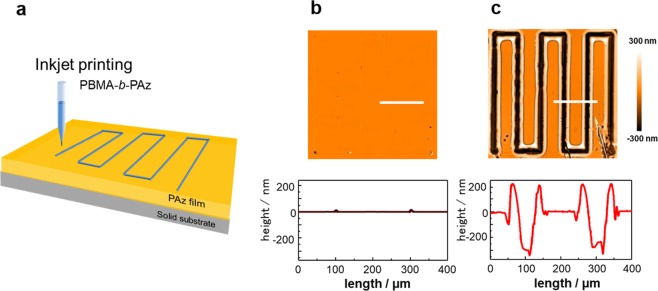
Instantaneous photoinitiated surface morphology change under optimized conditions. (**a**) Scheme of inkjet-printing drawing. (**b**) A surface topographical image for an initial as-printed film taken by WLIM; 2D image (upper) and cross-sectional height profile traced along the white line (lower). (**c**) The same measurement as B after UV irradiation (10 mW cm^−2^) at 90 °C for 30 s. A movie of the motion observed with an ordinary optical microscope is provided in Supplementary Movie 1.

Under optimal temperature conditions, the migrating motion became instantaneous following the photoisomerization progression (Fig. 3). At 90 °C (approximately 20 °C lower than the isotropization temperature (*T*_iso_)), the motion ceased over nearly the same time course as the photoreaction (20 s). Fig. 3b,c show surface morphology changes evaluated by white-light interferometric microscopic (WLIM) observations. Before UV-light irradiation, the surface profile indicated small protrusions of the printed line (Fig. 3b, lower). In contrast, after UV irradiation for 30 s, enormous surface deformations were observed (Fig. 3c). As shown in the images of b and c, the initial printed line was nearly invisible, and in contrast, the mass migration by the irradiation, gave a large trench formation approximately 300 nm in depth and 200 nm ridges approximately 200 nm in height on both edges. Using an ordinary optical microscope, the migrating motion could be monitored. In Supplementary Materials, a video showing real-time surface morphology changes under irradiation is provided (Supplementary Movie 1). As shown in the movie, the mass migration was initiated immediately after the UV-light irradiation.

The static contact angles of a glycerol droplet (*θ*_gly_) on the film surfaces of PAz and PBMA-*b*-PAz under UV-light irradiation at 90 °C were 85 ± 1.5° and 102 ± 1.1°, respectively (Table 1), indicating that the surface tension of the PAz surface was larger than that of PBMA-*b*-PAz. Thus, the migration direction occurred from lower to higher surface tension regions. In Supplementary Fig. 2, the *θ*_gly_ values at various temperatures under UV irradiation are indicated. At all temperatures, *θ*_gly_ on cis-isomerized PAz was smaller than that of PBMA-*b*-PAz. These facts strongly suggests that the driving mechanism of the above photoinitiated migrations is the surface tension driven flow by the Marangoni effect, as has been suggested in former studies using photopatterned amorphous polymer films upon heating above *T*_g_^12–16^. The rate of mass migration in this work was notably fast compared with those of these previous examples. This can be a particular feature for a system utilizing the photoinduced phase transition in liquid crystalline polymers.

After UV irradiation, once the trench was formed, the surface morphology was extremely stable. The valley structure was essentially unchanged, even at 130 °C for 7 days (Supplementary Fig. 5). This means that the resultant morphology is persistently maintained as far as a different polymer component exists on the free surface. If the polymer ink is removed from the surface, the capillary force due to the surface tension should flatten the surface. To confirm this, the printed ink was selectively rinsed from the surface-inscribed surface. In this experiment, PBMA instead of PBMA-*b*-PAz was printed on the PAz film surface. The PBMA homopolymer was also found to induce the mass migration, and with this polymer selective removal of the printed ink was possible because of the large solubility difference between PBMA and PAz in cyclohexane (Supplementary Fig. 6a). From the surface-deformed film of PAz, PBMA was selectively rinsed with cyclohexane at room temperature for 2 min. The rinsing did not affect the morphology at this stage. UV irradiation of this film at 90 °C resulted in a flat surface formation (Supplementary Fig. 6b). As indicated in Supplementary Fig. 5, the ridges were flattened after 7 day heating. In this way, the effect of capillary force was admitted as a very slow process when the ink exists on the top.

By evaluating the cross-sectional area of the height profile after UV-light irradiation, the volume of the ridge portions was roughly in agreement with that of the trench. This fact unequivocally indicates that the morphology change is due to mass migration. The volume of PAz film driven by the surface ink of PBMA-*b*-PAz (*V*_PAz_/*V*_PBMA-b-PAz_ (=*f*)) was roughly estimated by the areas of cross section profile. This can be regarded as the amplification factor. Under the same conditions (60 °C, UV: 10 mW cm^−2^ for 5 min), the value of *f* increased with film thickness in the same conditions. When the film thicknesses of PAz film were 350, 600, and 1000 nm, the *f* values were 58, 210, and 440, respectively. The thicker film provided the larger amplification factor.

### Features of the printing-assisted photo-driven process

**Figure 4:**
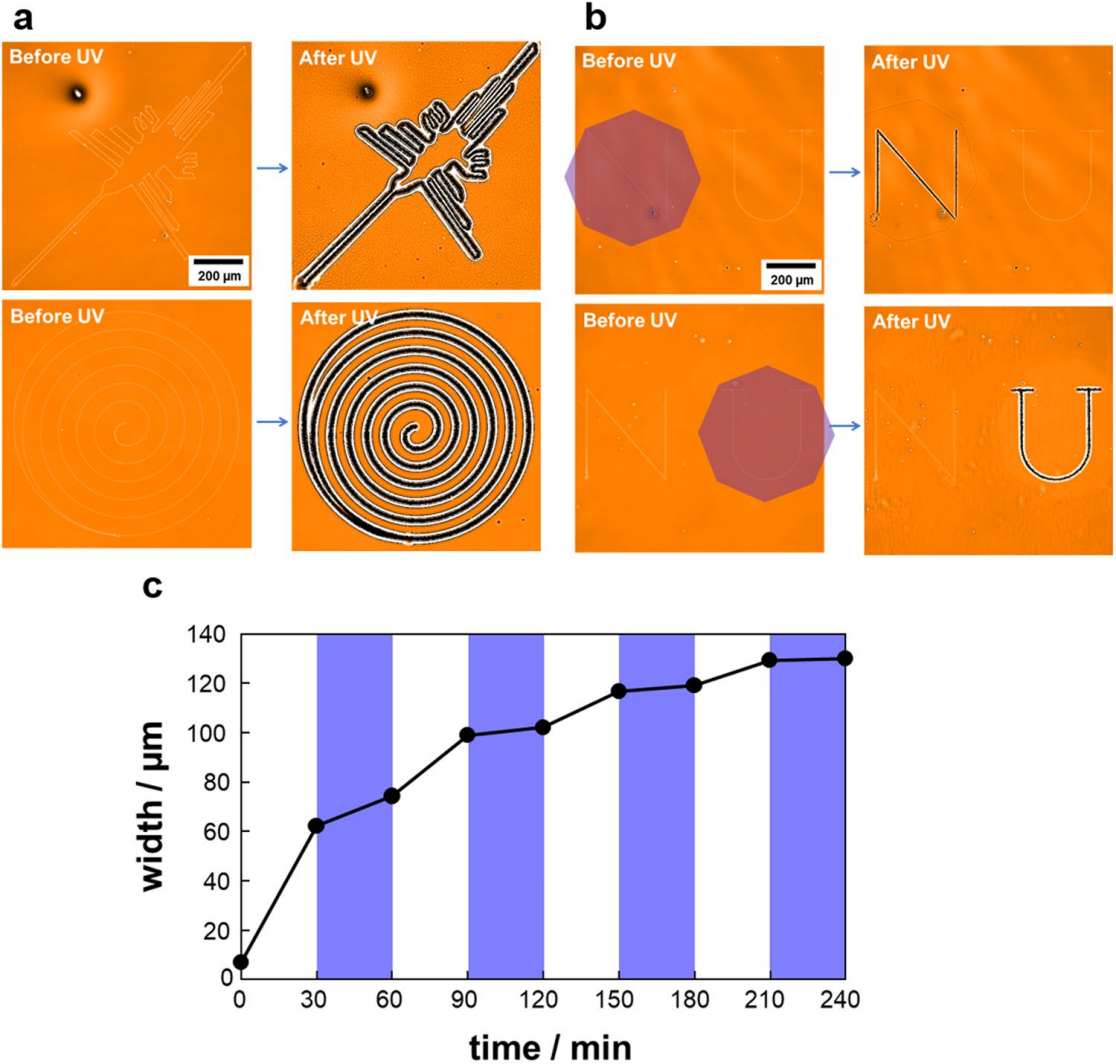
Variations in the photoinitiated mass transfer processes. (**a**) Various inkjet drawings leading to curved trenches observed by WLIM. The corresponding real-time motions are given in Supplementary Movie 2. (**b**) Two letters that are individually irradiated. Only the selectively irradiated letter within the octagonal area appeared. (**c**) On-off switching control of the transfer motion by alternating irradiation with UV and visible light.

The inkjet printing briefly enables arbitrary drawings. Figure 4a displays examples of curved drawings (Nazka humming bird (upper) and spiral circle (lower)). The real-time motions are given in Supplementary Movie 2. The particular features of the photoinitiated mass migration process, instead of the heat-assisted one, can be identified by two aspects. First, irradiation is remotely achievable, which enables a selective inscription. In Fig. 4b, two letters, N and U, were inkjet printed. The figures of N and U were inscribed independently with UV irradiation on the left- and right-hand sides of this field, respectively. This type of spatial selection is difficult in heating processes. Second, on-off switching of the mass migration is readily performed. Figure 4c shows the time course of the linewidth upon alternative irradiation with UV (365 nm, cis-azobenzene content: 76%) and visible (436 nm, cis-azobenzene content: 11%) light at 75 °C. The linewidth evolution progressed under UV-light irradiation, and slowed under visible-light irradiation that switches back to the smectic A phase. This control could be achieved repeatedly. This fact indicates that the linewidth can be controlled arbitrarily by the UV irradiation time. The slowing by visible light was not sharply achieved because several seconds were needed to alter the irradiation wavelength. In the heat-melt process, such prompt switching should also be difficult. The above two features are expected to expand the possibilities of the Marangoni flow process for the formation of engineered surfaces.

### Evaluation of polymer ink spreading

**Figure 5:**
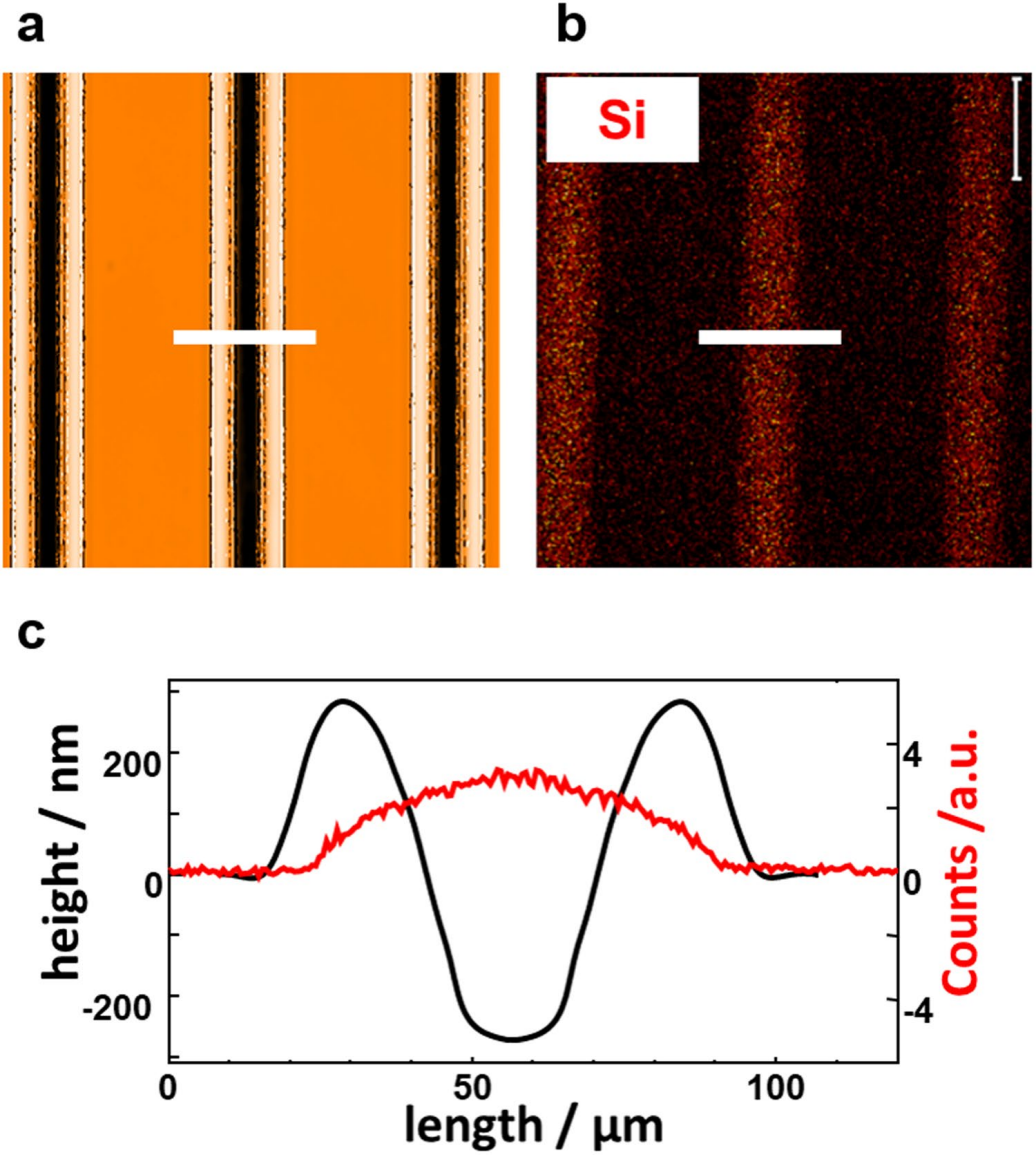
Observation of the distribution of the top of a printed line after mass transfer. (**a**) Topographical WLIM image for a PAz film surface after UV irradiation at 60 °C. (**b**) TOF-SIMS image exhibiting the distribution of Si ions. (**c**) compares the surface morphology outline (black) and the corresponding Si ion distribution profile (red) along the white lines of the upper figures.

To gain further insight into this dynamic process, we attempted to observe the lateral distribution of the printed polymer ink after the mass transfer. The evaluation by a surface profile measurement such as AFM was difficult because the surface deformation was too large to detect the subtle embossment of the polymer ink. A rough estimation after mass migration by AFM provided approximately 5 nm as thickness of the spread ink. To obtain the accurate spreading behavior, a time-of-flight ion secondary ion mass spectroscopy (ToF-SIMS) measurement was made^6^ using PDMS-*b*-PAz instead of PBMA-*b*-PAz. With this polymer, elemental Si can be the probe for 2D mapping of the ink distribution. Fig. 5a,b show a surface profile obtained from WLIM measurement and a ToF-SIMS image after sufficient UV irradiation, respectively. In Fig. 5c, a profile indicating the abundance of elemental Si (red line) is superimposed with the height profile in the regions of the white bar in a and b. As revealed, the Si was distributed almost between the edge regions of the two ridges. Therefore, it was confirmed that the printed polymer line laterally expanded over the two ridges as a consequence of the mass migration.

This fact should indicate that the amount of mass migration depends on the amount of printed ink. When the ink is fully spread, the migration motion should cease. In fact, a larger ink amount resulted in a larger mass migration until this spreading level was attained (Supplementary Fig. 7).

### Thermally induced mass migration

**Figure 6:**
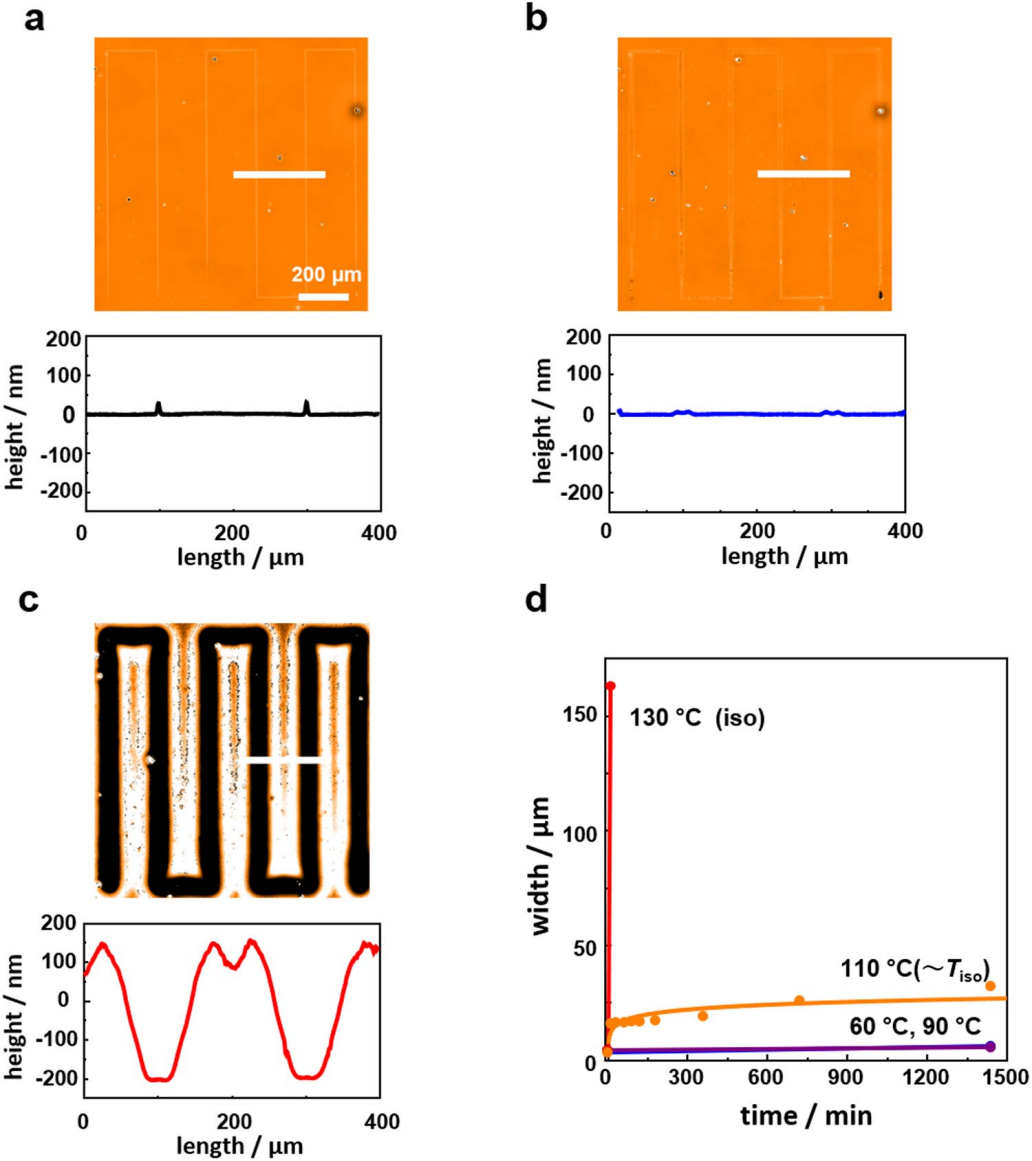
Thermally induced mass migration from a printed line. (**a**) A WLIM topographical image (upper) of an as-printed PAz film and cross-sectional height profile showing the height of the printed PBMA-*b*-PAz along the white line (lower). (**b**) The same measurement after annealing at 110 °C for 24 h. Note that only lateral spreading of the printed polymer line was observed without the formation of a trench. (**c**) The same measurement after annealing at 130 °C (isotropic state) for 10 min. (**d**) Time-course profiles of linewidth at 60 or 90 °C, 110 °C, and 130 °C.

The thermally induced transition in the dark was explored in addition to the photoinduced transition because more precise understandings were expected to be obtained. Figure 6 shows the evolution of linewidth in the dark at various temperatures. Essentially no morphology change was induced below 90 °C (a). At 110 °C (T ≃ *T*_iso_), the line showed some expansion, but no dip formation was observed; only the printed ink showed spreading on the surface (b). A drastic change was observed at temperatures above *T*_iso_. At 130 °C, a clear mass migration quickly proceeded to form a trench with a ridge on each both side (c). Thus, in both the photochemical and thermal cases, the effect of the transition from a smectic A to isotropic phase on the mass migration was enormous.

In the thermal transition, the reduction in viscosity was precisely evaluated. The shear viscosity measurements at 90 °C (smectic A state) and 130 °C (isotropic state) revealed that the viscosity of the PAz was reduced up one to three orders of magnitude by the thermal phase transition to the isotropic state (Supplementary Fig. 4c). As mentioned previously, the quantitative estimation of viscosity change by the photoinduced phase transition was difficult due to the measurement problem. However, considering the fast mass migration observed, it is anticipated that the viscosity reduction is of the same magnitude (Fig. 3).

The surface tension was evaluated by the static contact angle of glycerol (*θ*_gly_, Supplementary Fig. 2). The *θ*_gly_ values on PAz and PBMA-*b*-PAz at 130 °C were 93.3 ± 1.8° and 101 ± 2.5° (Table 1), respectively. As shown in Fig. 4B, at 110 °C, a spreading of PBMA-*b*-PAz ink was observed without trench formation. During 24 h, a lateral expansion of ink width was observed from 5 μm to approximately 30 μm at this temperature (Supplementary Fig. 8). When this film was subsequently heated to 130 °C, a large mass transfer immediately occurred to form a clear trench. In this case, the bottom of the trench was flat for a distance of 30 μm; although the lowest height was not that of the substrate level. This means that the mass transfer occurred at the boundary edge of the printed ink and an even surface tension allows the surface to remain flat (C). Until this point, all the polymer inks consistently possessed lower surface tension than that of PAz. When a polymer ink with higher surface tension (poly[4′-6-(methacryloyloxy)hexyloxy-4-cyanobiphenyl] (PCBMA)) was used (*θ*_gly_ on PCBMA = 80.3 ± 1.4° <*θ*_gly_ on PAz = 93.3 ± 1.8°) (Table 1)), the direction of the mass migration was inversed. In this case, heating above *T*_iso_ resulted in a migration to form a single embossment with two receded trenches on both sides (Supplementary Fig. 9). These data obviously support the interpretation of the Marangoni flow as driving mechanism.

## Discussion

### Revisiting amorphous SRG systems by patterned light irradiation

Since 1995, the optical generation of surface relief gratings (SRGs) on azobenzene-containing polymer films has been a subject of extensive study and discussion^40–45^. A unified explanation of the mass transfer behavior has not yet been given^42^. At least, little attention has been paid to the non-uniformity of the surface tension of the top layer. In this regard, the work by Viswanathan *et al*.^43^ at an early stage of photoinduced SRG study (just a few years later than the discovery of SRG phenomena) is to be noted. They showed the significant role of the free surface side in the SRG generation. When an azobenzene-containing amorphous polymer is covered with a molecular-level film by the layer-by-layer (LbL) electrostatic deposition on the top, the SRG formation is abruptly hindered. It has been explained that the disruption of surface topographical change by the overlaid LbL layer is the reason for hindrance of the mass transfer. Additionally, it seems reasonable to consider the effect of a surface tension modulation due to an uneven distribution of trans/cis azobenzene isomers in the uncovered azobenzene polymer film. Actually, Kim *et al*.^15^ have discussed the mass migration process of an azobenzene-containing polymer film induced by the Marangoni effect. However, it is also evident that the surface-tension driven motion alone cannot explain the polarization effect of light, e. g., the mass transfer occurs along the electric vector of LPL^40–45^. Anisotropic photoinduced fluidity can also affect the migration^46^. The SRG formation in amorphous polymer films requires careful interpretations, but it seems necessary to consider the contribution of Marangoni flow effect.

### Implication for SRG formation in liquid crystalline polymer films

The mechanism of SRG formation in SCLCP films upon patterned irradiation^47–50^ should be closely related to the present system. The motions involved in the previous studies are associated with the photochemical phase transition of azobenzene polymers^34,36,38^. The difference is whether the irradiation is achieved with patterned light (former SRG work) or overall exposure (this work). In conclusion, we assume that the Marangoni effect also plays the important role in the previous SRG formation systems for the following reasons. First, the migration motion occurs most efficiently at the boundary edge of the photomask patterns^48,50^, which is consistent with the observations by Kim *et al*.^15^ Second, the mass migration does not exhibit a dependence on the polarization of the irradiating light^49^. The mass migration behavior is very similar to the present investigation in terms of the effect of light exposure dose, an insensitivity to polarization, the surface shape possessing a receded trench with ridges along the edges. Possibly, the former SRG systems are driven in large part by the surface tension gradient formed by the patterned light. In patterned irradiation^39,47,48^, the trans/cis isomerization ratio patterns are formed by patterned irradiation, thereby, both the surface tension and rheological properties of interior parts are changed simultaneously. In contrast, in our present approach by inkjet printing, only the surface tension modification is obtained under uniform UV irradiation. Therefore, our approach is more favorable to precisely evaluate the Marangoni effects.

### Significances and outlook

Inkjet printing is the key tool widely applied in printed electronics. This work demonstrates a new method of utilizing inkjet printing. The base films for printed electronics, even when flexible substrates are used, do not exhibit large deformations at local-area levels, otherwise disconnections of the printed electric circuit can occur. This work newly proposes highly deformable base film systems, where the polymer chain dynamics (rheology) are on the same order of the ink material. In this sense, the approach shows a marked difference from other well-conceived applications of inkjet printing.

The significant role of the free surface in polymer films is re-realized. With regard to molecular orientation, the free surface strategy works well for SCLCPs^21–28^, as mentioned in the introductory remarks. There are other important examples. Surface-segregated monolayers have been introduced for orientation control of semiconducting polymers^51,52^, and the modification of a free surface or covering is important for the orientation control of the separated microphase structure in block copolymer films^53^. Proposals by Ellson *et al*.^12–16^ and this work further present a new direction to induce surface morphing by utilizing the Marangoni effect.

UV irradiation using a low-cost light source (mercury lamp or LED lamp) and a simple optical setup have advantages. This is in contrast to the Marangoni flow-induction using a laser beam scanning system^17–19^. Furthermore, compared with the heating procedure results, the morphology formation is attained remotely and selectively only at target portions, and irradiation light dose controls the extent of mass migration (Fig. 4). These facts enable the designed control of morphology inductions by photoirradiation operation. Such a light-regulated Marangoni flow process has not been reported before.

Expected applications of the present method will be found in microfluidics technology, efficient light extraction or moth-eye surfaces for display panels, distributed feedback lasing devices etc. Here, the fabrication is achieved as designed by inkjet printing instead of photolithography. For the use of microfluidics, the channel wall in this work is covered with a lower surface tension polymer. A suitable liquid may be efficiently guided on a chip device not only by the channel shape but also by a surface energy contrast. Furthermore, the print-assisted deformation can be concave or convex, depending on the relative surface tensions of the ink material and the base polymer. This work is focused on concave (trench) formation, but the same strategy can be applied for convex (embossment) formation using a different polymer ink with a higher surface tension (see Supplementary Fig. 9).

To gain a precise understanding, more explorations are needed using a variety of base polymers and ink materials. Such efforts are also required to evaluate the applicability of this strategy. The accumulation of data along this line is currently in progress. At the same time, the presentation of a proper physical model describing the features during the evolution of time and the morphology induction is essential for precise explanations of the present systems. Efforts in these directions are also currently underway.

In conclusion, we newly demonstrate the photoinduction of surface morphology spatially directed using a sub-femto-liter inkjet printing technique. Using a lower-surface-tension polymer as the ink, the mass migration of a liquid crystalline photoresponsive base polymer film is initiated by irradiation with UV light. This action results in a crater or trench formation accompanied by an isotropic isothermal phase transition of the liquid crystal. The mass transfer behavior of the polymer material is reasonably explained by the Marangoni effect. The inkjet printing technique is extensively utilized and is in development in the field of 2D patterning on substrates, especially for printed electronics with high spatial accuracy. Therefore, we anticipate that the proposed strategy will provide a new type of platform possessing a large potential to be comparable with other widely known microfabrication methods such as photolithography, soft lithography^54^, block copolymer lithography^55^, photoinduced surface relief grating^39–45,47–50^, surface wrinkling^56^ etc. On the other hand, much attention has already been paid to azobenzene-containing SCLCPs. They show intriguing photoresponsive effects and functions^33,57,58^ such as holographic optical recording, surface photoalignment of liquid crystals, photoinduced phase transitions, surface relief grating formations, and photoinduced deformations that allow macroscopic optical actuators. This phototriggered Marangoni effect can be recognized as an intriguing new optical effect in addition to the above widely recognized photoprocesses.

## Methods

### Chemical Materials

Unless stated otherwise, all reagents and solvents were obtained from commercial suppliers and used as received. Polymer materials abbreviated as PAz, PBMA, PBMA-*b*-PAz, PDMS-*b*-PAz, and PCBMA were synthesized by the atom transfer radical polymerization (ATRP) method according to the previous papers listed in Table 1.

### Measurements

The Molecular weight and the molecular weight distribution were measured by gel permeation chromatography or high-performance liquid chromatography (Showa Denko K. K., Japan). The liquid crystal transition temperatures and glass transition temperatures were evaluated by DSC (TA Instrument Q200, USA) and a polarized optical microscope (BX-51, Olympus Corp., Japan).

The film thicknesses were measured with a white-light interferometric microscope (WLIM, BE-S501, Nikon Corp., Japan). The static contact angles of glycerol and water on the polymer films were obtained using a CA-XP (Kyowa Interface Science Co. Ltd., Japan) at various temperatures. The UV-Vis absorption spectral measurement was performed via UV/Vis absorption spectroscopy (Agilent 8453 spectrometer, Agilent Technologies, USA). The content of cis-azobenzene in the photostational state after irradiation with 365 nm and 436 nm light was determined spectroscopically by comparing absorbances at 370 nm and at 308 nm (isosbetic point)^47^.

A mercury-xenon lamp (Sanei Electronics Supercure 203S) was used for the light source. The 365 nm and 436 nm lines were selected by passing through combinations of glass filters of UV-35/UV35D (Toshiba) and 3–73 and 6–67 (Corning), respectively.

Steady flow viscosity measurements of the PAz polymer were performed at controlled temperatures using a MCR-301 corn-plate rotatinal rheometer (Anton Paar GmbH, Austria). The viscosity measurements under UV irradiation were achieved by introducing the light with an optical fiber to the rear side of the plate (see Supplementary Fig. 4).

The morphological changes were observed by WLIM (BE-S501, Nikon, Japan) or AFM (MFP-3D, Asylum Research, UK). The real time morphological change observations were performed by an optical microscope (BX-51, Olympus Corp., Japan) equipped with a light irradiation apparatus.

The ToF-SIMS measurement was performed with a PHI TRIFT V nano TOF (ULVAC-PHI Inc., Japan) at Aichi Center for Industry and Science Technology in Toyota city. In this measurement, the 30 kV Bi_3_^+^ primary ion beam (100 nm diameter) under vacuum irradiated an area of 150 × 150 μm with pixel resolution of 256 × 256.

### Sample preparations

The films were prepared by spin coating (Mikasa) from toluene solutions (typically 7.0 wt% PAz in toluene) onto quartz substrates.

The inkjet printing was performed using a subfemto-liter inkjet printing apparatus (SIJ Technology Inc., Japan). The arbitrary line drawing was achieved as programmed in a computer.

The polymer inks were printed on PAz films. The ink solutions were prepared with 0.5 wt% of polymer in mixed solvent of *o*-dichlorobenzene and *N*-methyl-2-pyrrolidone (30:1 by volume). UV-light irradiation (365 nm) was performed with an Hg-Xe lamp (UV supercure 203s, Sanei Electronic Co., Japan) at 10 mW cm^−2^, exposing the inkjet-printed PAz surface at a controlled temperature.

## Supplementary information

Supplementary Movie 1

Supplementary Information

Supplementary Movie 2

## Data Availability

The data that support this study are available within the article and its Supplementary Information files or available from the authors upon request.
