# Supplementary material for: Photoinitiated Marangoni flow morphing in a liquid crystalline polymer film directed by super-inkjet printing patterns
Source: Sci Rep. 2019 Feb 22;9:2556. doi: 10.1038/s41598-019-38709-1 (PMC6385296; doi:10.1038/s41598-019-38709-1)
Supplement: Supplementary file 2 — Supplementary Information [file 41598_2019_38709_MOESM2_ESM.pdf]

Supplementary Information for

Photoinitiated Marangoni flow morphing in a liquid crystalline polymer film directed by super-inkjet printing patterns

Issei Kitamura, Kazuaki Oishi, Mitsuo Hara, Shusaku Nagano*, Takahiro Seki*

Contents:

Supplementary Figures 1 – 9.

Explanations for Supplementary Movies 1 and 2.

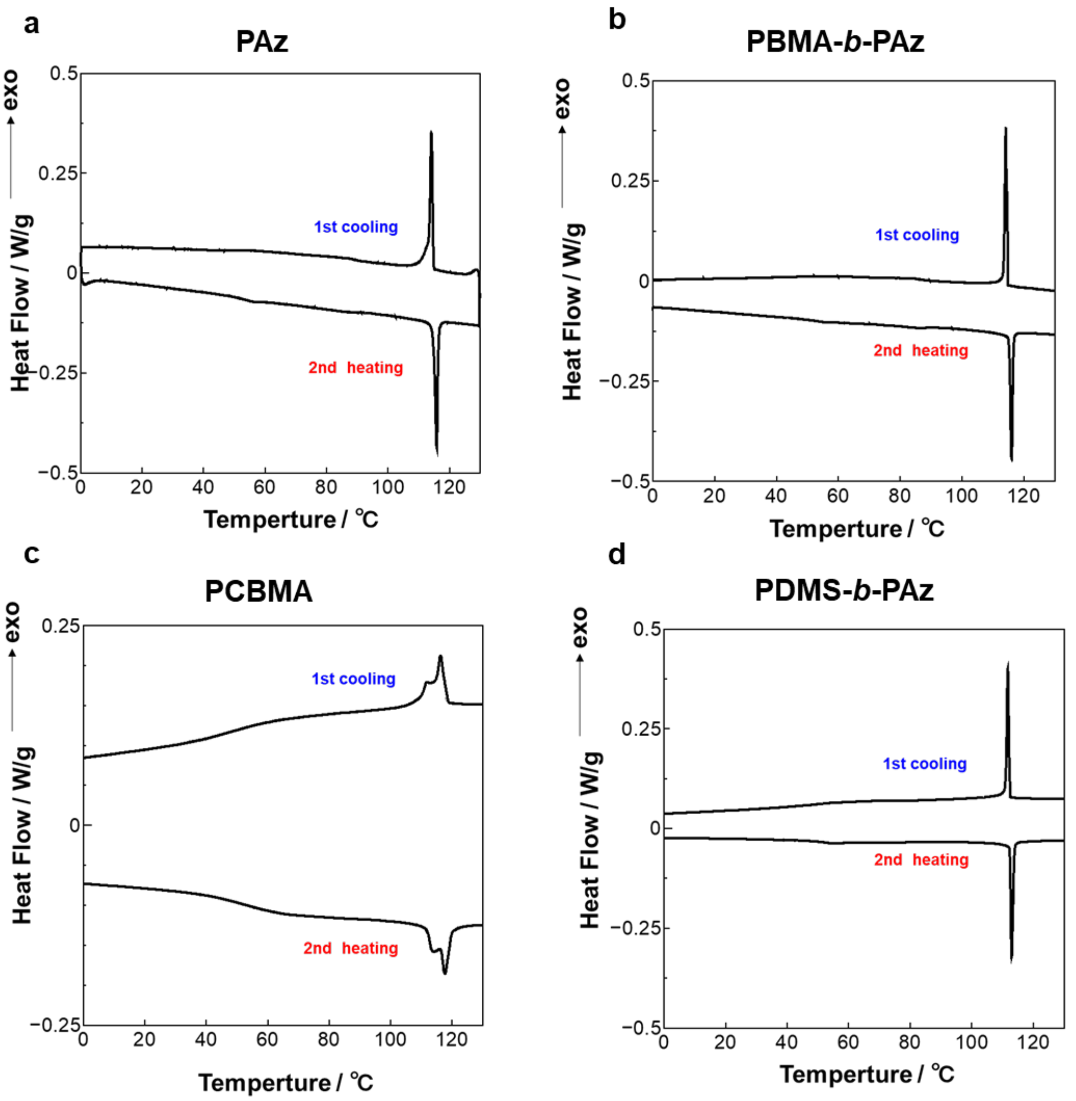

Supplementary Figure 1 DSC profiles of the liquid crystalline polymers investigated in this work. The phase transition temperatures are listed in Table 1 in the text.

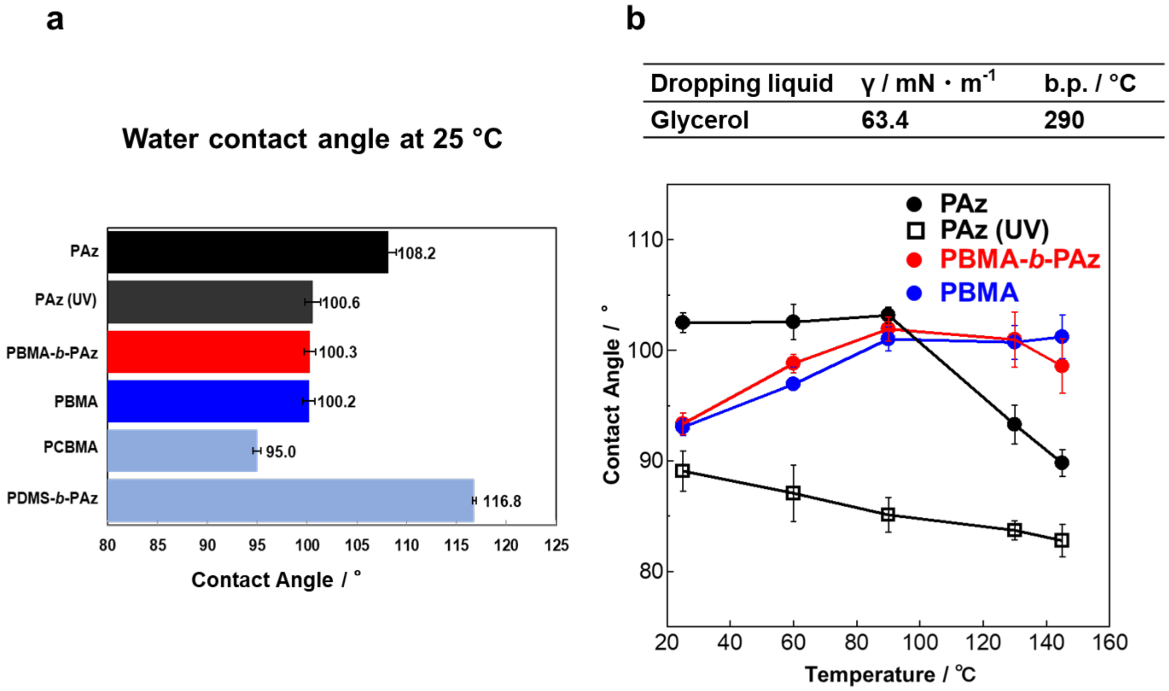

Supplementary Figure 2 Contact angle measurements. (a) The contact angle of a water droplet on various polymer film surface. (b) The contact angle of glycerol (θ_{gly}) on polymers at various temperatures.

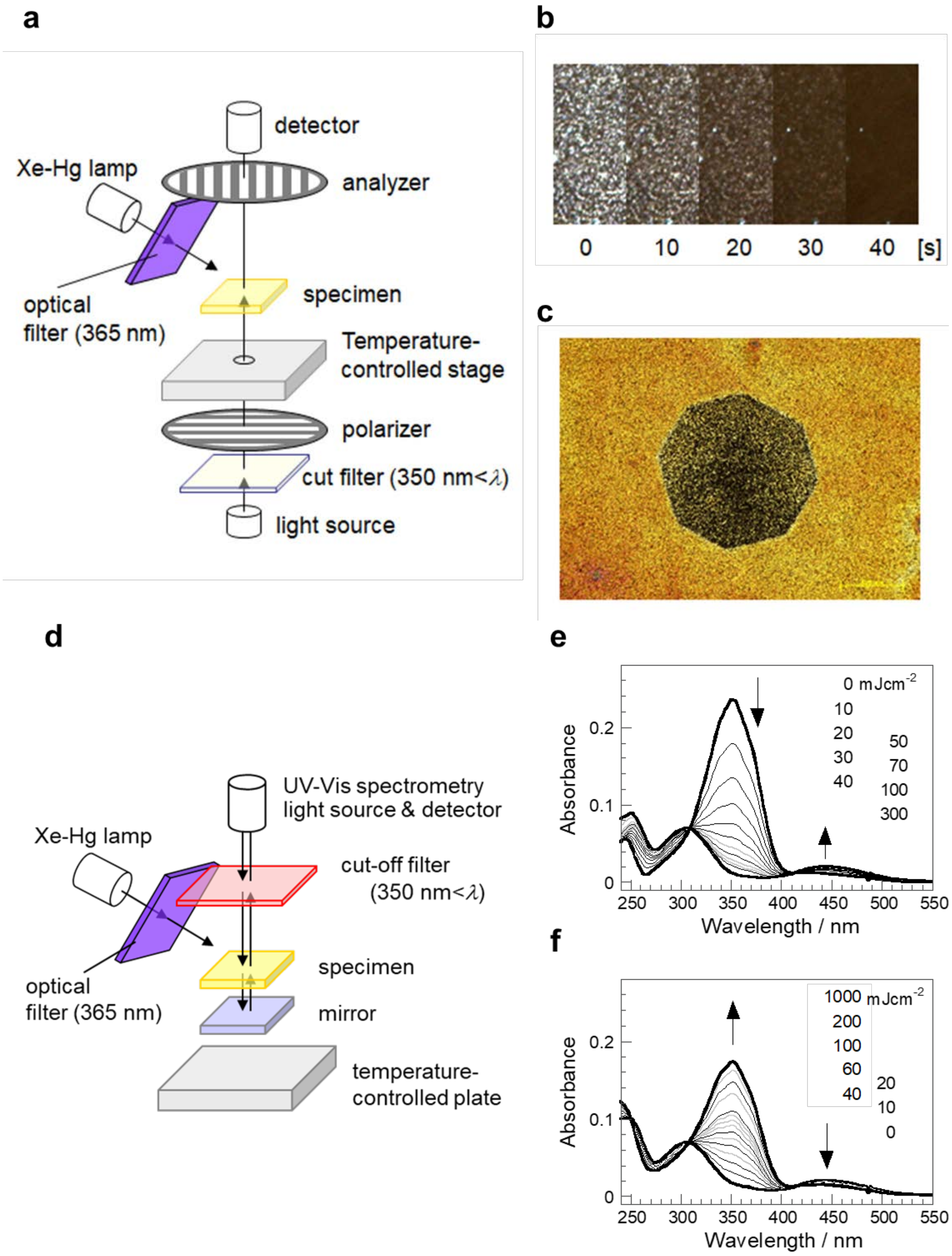

Supplementary Figure 3 Photinduced phase transition of a PAz film. (a) Polarized optical microscopic (POM) images showing the smectic A to isotropic phase transition of PAz film by irradiation with UV light (0.3 mW cm^{-2}) at 80 °C (changes in the POM image with time (b) and spot irradiation (c)). (d) UV-visible absorption spectral changes upon 365 nm (e) and 436 nm (f) light irradiation. Data are taken from reference 39 in the text.

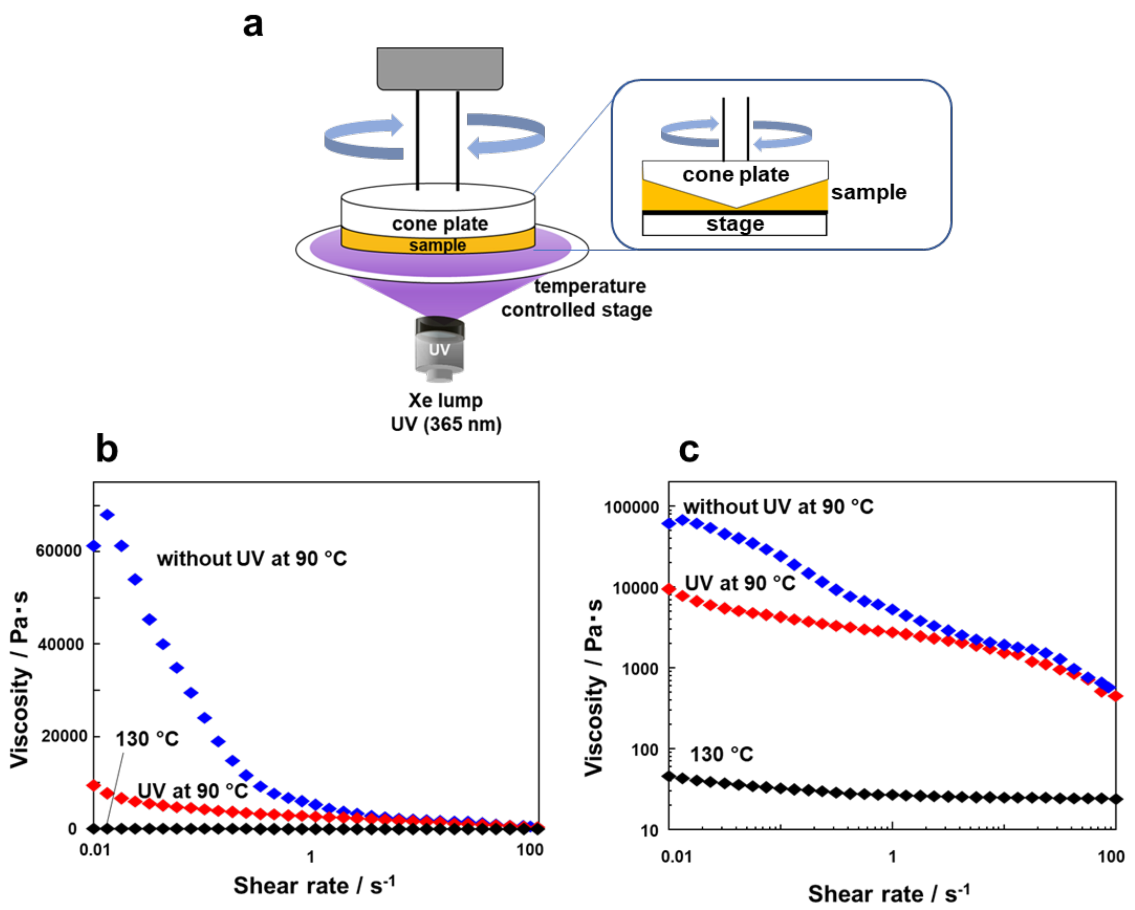

Supplementary Figure 4 Steady flow viscosity measurement without and without UV irradiation. (a) Schematic of viscosity measurement apparatus under UV-light irradiation. The light was introduced from the bottom of the table of viscometer. (b) Viscosity of PAz as a function of share rate. Blue and red diamonds corresponds to measurements made without and with UV irradiation at 90 °C. Black diamonds are data taken at 130 °C, isotropic state of PAz. (c) The same data displayed in the logarithm scale in the viscosity axis.

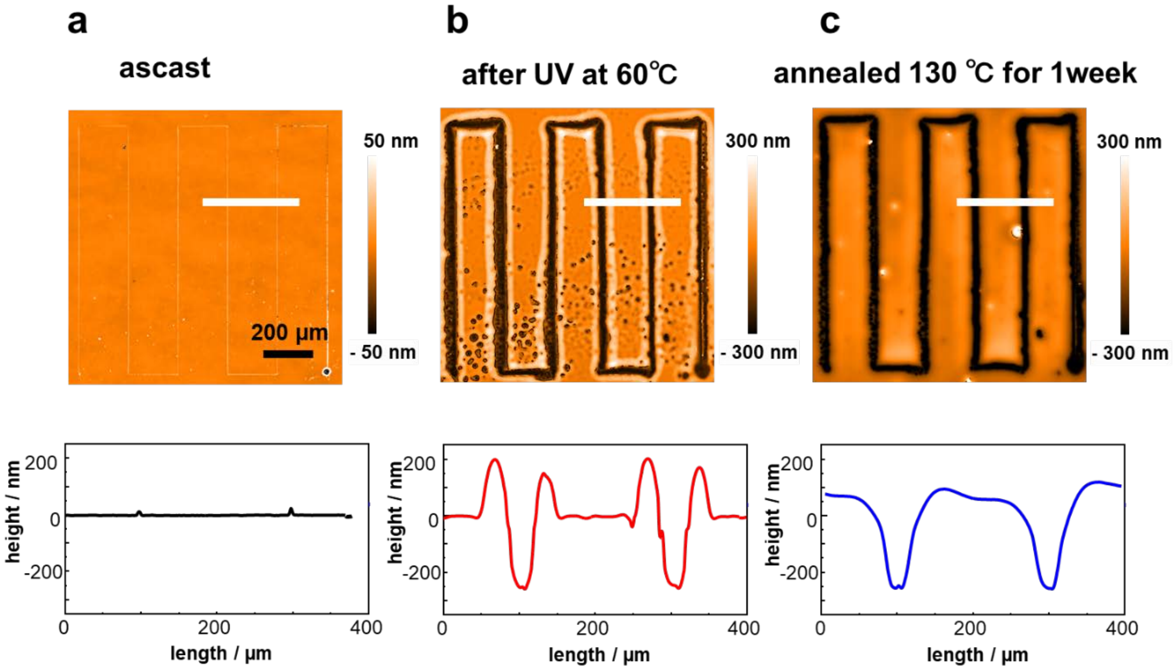

Supplementary Figure 5 Stability of the photogenerated morphology. Surface morphologies evaluated by WLIM. (a) as-cast, (b) after UV irradiation at 60 °C, and (c) annealed at 130 °C for 7 days from the state of b. Note that the trench depth is essentially kept unchanged although the ridges are smoothened after annealing at 130 °C for 7 days.

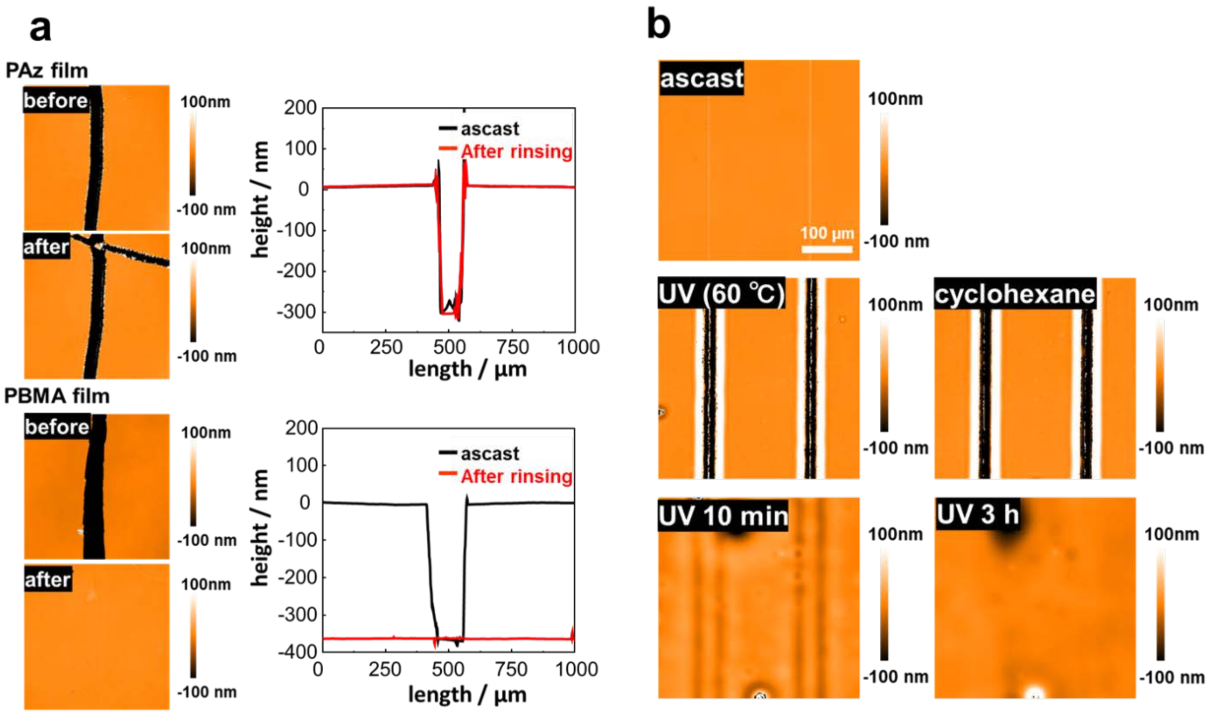

Supplementary Figure 6 Selected removal of the printed polymer ink and the morphology change. (a) Solubility tests showing that PAz is not dissolved in cyclohexane while PBMA fill was fully dissolved in this solvent as revealed with scratched film of PAz and PBMA films. (b) Morphologies of an as-printed film with PBMA, the inscribed film by UV-light irradiation (UV (60 °C)), after rinsing with cyclohexane selectively removing PBMA ink on the top, and UV irradiation with 10 min and 3 h at room temperature. All images were taken by WLIM.

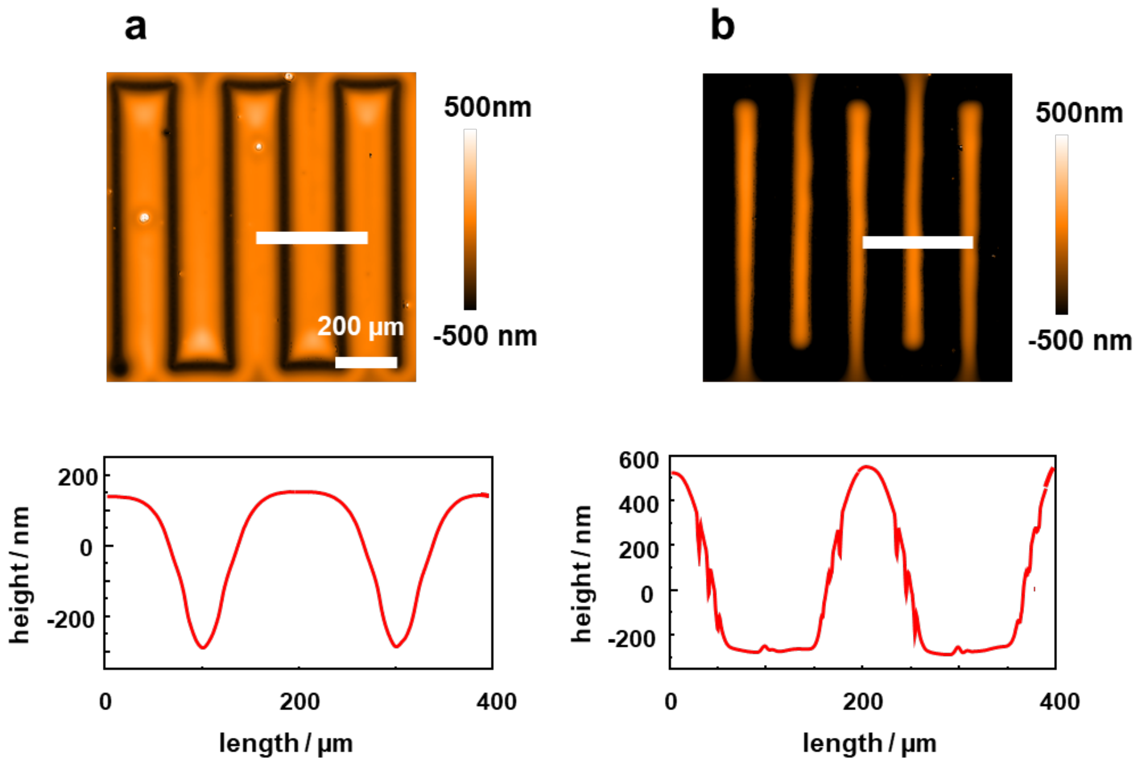

Supplementary Figure 7 UV-initiated mass transfer behavior of a PAz film induced by different amounts of polymer ink. (a) WLIM images of an as-printed PAz film (upper) and that after UV light irradiation (1 mW cm^{-2}) for 12 h from the printed line with 70 nm. (b) The same procedures from the printed line with 300 nm height.

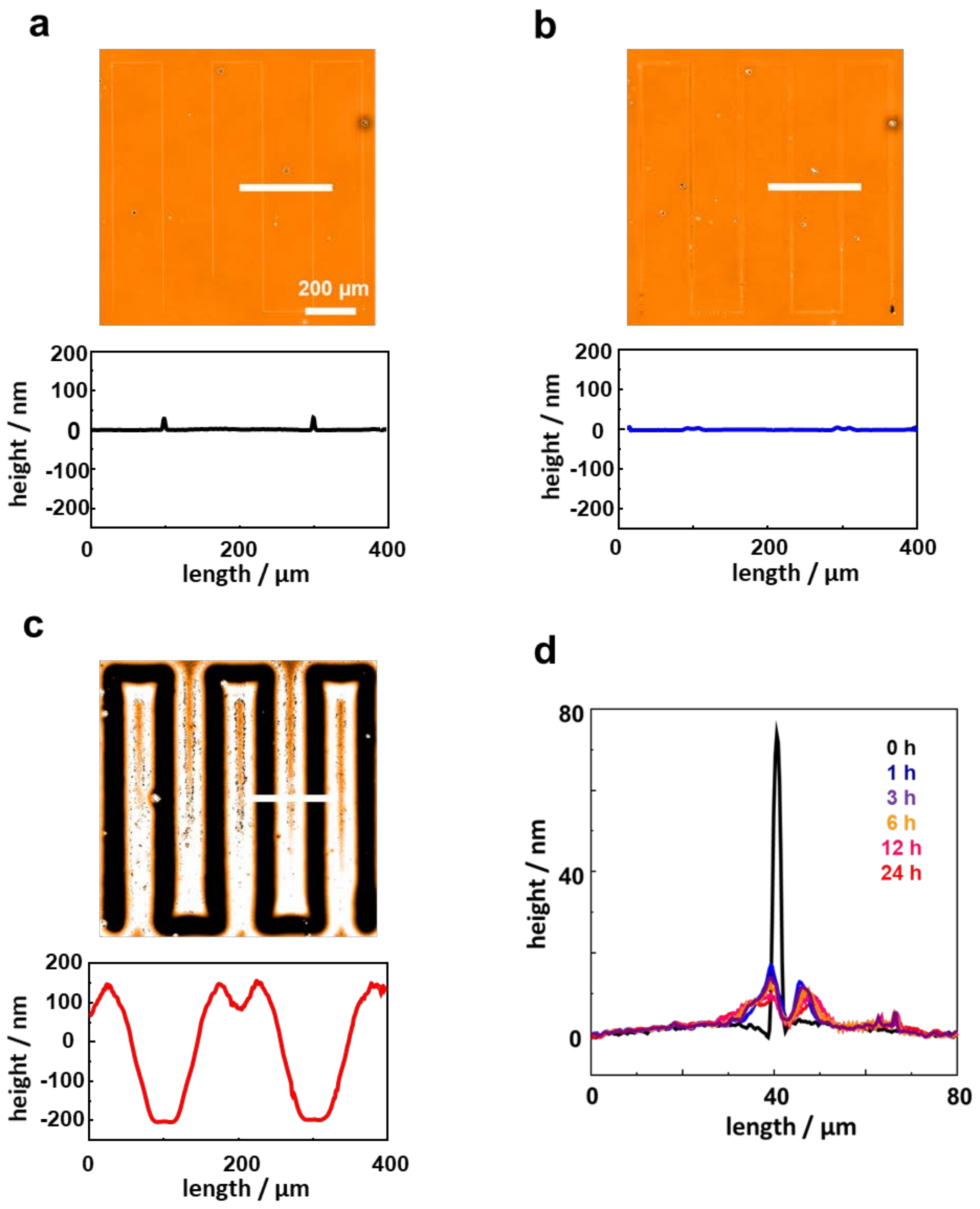

Supplementary Figure 8 Mass migration from a wide line. (a) WLIM images (upper) and a corresponding surface profile (lower) along the white line. (b) The same measurement after annealing at 110 °C for 24 h. (c) The same measurement after annealing at 130 °C starting from the state of B. (d) The magnified surface profiles of the printed line monitored at 110 °C.

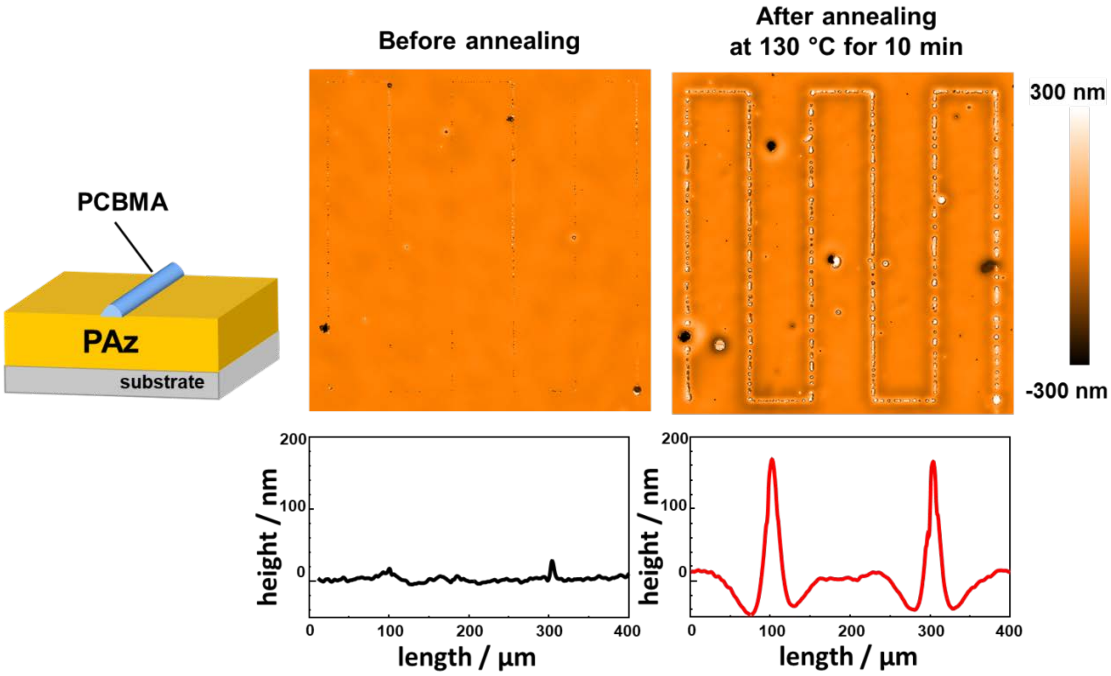

Supplementary Figure 9 Ridge formation at the center triggered from a PCBMA line on a PAz film. Note that the migrating flow occurs towards the printed line with the higher surface tension.

Supplementary Movie 1. Real-time optical microscopic observation of photoinduced morphology change at the inkjet printed drawing corresponding to Fig. 2b to Fig. 2c.

Supplementary Movie 2. Real time optical microscopic observation of photoinduced morphology change at the inkjet printed drawing corresponding to Fig. 3a (upper).
